# Supplementary material for: Factors shaping network emergence: A cross-country comparison of quality of care networks in Bangladesh, Ethiopia, Malawi, and Uganda
Source: PLOS Glob Public Health. 2024 Jul 23;4(7):e0001839. doi: 10.1371/journal.pgph.0001839 (PMC11265678; doi:10.1371/journal.pgph.0001839)
Supplement: S1 Table — (DOCX) [file pgph.0001839.s001.docx]

**S1 Table:** Role of Key QCN Actors in Four Pathfinding Countries

| **Key Stakeholders** | **Roles in different countries** | | | |
| --- | --- | --- | --- | --- |
|  | **Bangladesh** | **Ethiopia** | **Malawi** | **Uganda** |
| **Government Actors** | | | | |
| **Ministry of Health** | QCN placement in Quality Improvement Secretariat (QIS).   - National government regulatory function - Played leadership and coordination role. | - QCN placement in Ethiopian Federal Ministry of Health. - Coordination and implementation role: harmonized partners and regional health offices to implement the roadmap but also lead the network and prioritized the agenda. - Co-designed with Institute for Healthcare Improvement (IHI) three-pronged approach, Ethiopia Health Care Quality Initiative (EHCQI) to accelerate health system improvement nationally. | QCN placement in Quality Management Directorate (QMD).   - Played the role of technical partner and involved in the development of strategic documents including the QCN roadmap, guidelines, conducting assessments, capacity building and supporting districts (subsequent reduced interactions). - Also involved in coordination and resource mobilization. | QCN placement within Department of Quality Assurance, renamed in 2019 to the Standards Compliance Accreditation Patient Protection (SCAPP) in 2019.   - Played roles in policy and guidance, coordination and technical support, resource mobilization, supervision and monitoring. - Actual implementation of QCN activities, however, was through public health facilities. |
| **UN Agencies** | | | | |
| **UNFPA** | - Technical capacity development. - Working on reproductive health and MPDSR. | - Provided both financial and technical support. | - Played the role of technical and implementing partner. They also largely provide funding to support RHD activities but also provide funding to QMD. - They supported the development of policies and strategic documents at the national level. - They worked with implementing partners to support QCN efforts which have included the quality of care baseline assessments, development of QoC tools, capacity building efforts and participate in the coordination meetings. - Member of the QM Technical Working Group. | - Technical, implementing and donor partner. |
| **UNICEF** | - Technical, implementing and funding partner. - Initially funded by BMGF, later Global Affairs Canada and the UN Emergency Fund for the Rohingya in Cox’s Bazaar. | - Provided both financial and technical support. | - Provided both financial and technical support. - Funded by Korean International Cooperation Agency (Thyolo). | - Technical, implementing and donor partner (also through Ips). |
| **World Bank /Global Financing Facility (GFF)** |  |  |  | - Financial support: supported the Uganda Ministry of Health’s QCN work through financing URM-CHIP (Uganda Reproductive, Maternal, and Child Health Improvement Project)/ Results-based financing (RBF) at all levels from national to facility level. |
| **World Health Organization (WHO)** | - Initially technical partner only (but since late 2021, shifted to coordination role and limited funding, e.g. salary of national consultant). | - WHO was working for the establishment of the coordination platform, and convincing, gathering and engaging other partners (Coordination). - Supported QCN learning facilities at the ground level. - Served as a link to the WHO headquarters and the QCN at the global level. | - Provided technical and financial support. - Since 2021, shifting from a supportive and monitoring role to a more leading role . | - Coordination and technical support (standards, etc.). - Funding implementation of some QCN sites e.g Hoima and Luuka districts. - Served as a link to WHO headquarters and QCN at the global level. |
| **Other Implementing Partners** | | | | |
| **Bill & Melinda Gates Foundation** | - Played funder role at QCN global level (through funding to WHO and UNICEF). | - Played funder role at QCN global level (through funding to WHO and UNICEF). | - Played funder role at QCN global level (through funding to WHO and UNICEF). | - Played funder role at QCN global level (through funding to WHO and UNICEF). |
| **GIZ** |  |  | - Provided technical support to the QMD at the national level and at the local level, supporting implementation of QCN activities in one district (Lilongwe) only. |  |
| **Institute of Healthcare Improvement (IHI)** | - Provided technical assistance through Save the Children. - Maintained liaison with global level. | - Implementing partner until 2019. |  | - Provided technical guidance on QI matters though was not a direct network member. |
| **Save the Children** | - Technical and implementing  partner. - Consortium  partnership with IHI. | - Provided technical support as a member of the larger national quality technical working group (TWG). | - Provided technical support. - Participated in QCN coordination meetings. - Reviewing the QCN roadmap and sharing best practices in quality of care. | - Implementing in selected districts. - Funded by USAID. |
| **University Research Co. (URC)** | - Technical support, particularly in training of health workers. |  |  | - Technical support, funding support and actual implementation (previously, with USAID support). |
| **USAID** | - Provided technical and financial support through Save the Children. | - Provided technical and financial support  through its affiliated partners, Transform PHC and Transform HDR. | - Provided technical and financial support. - Participated in QCN coordination meetings and reviewing the Malawi QCN roadmap. Also provided financial support to districts (e.g. Mangochi and Zomba) organizations like Organized Network Services for Everyone (ONSE). - Involved in the development of assessment tools for the QoC standards among other organizations. | - Technical and implementing partner; supports implementation through regional partners FHI 360 and RHITES. - Later (2021), worked with MoH to revitalize the QCN Technical Working Group at the national level with new meetings and guidance. |
