# Supplementary material for: Factors shaping network emergence: A cross-country comparison of quality of care networks in Bangladesh, Ethiopia, Malawi, and Uganda
Source: PLOS Glob Public Health. 2024 Jul 23;4(7):e0001839. doi: 10.1371/journal.pgph.0001839 (PMC11265678; doi:10.1371/journal.pgph.0001839)
Supplement: Attachment — Submitted filename: QCN emergence_reviewer responses.docx [file pgph.0001839.s005.docx]

Dear Dr. Hall-Clifford,

Thanks for the opportunity to revise and resubmit our manuscript. We are happy to see that the reviewers find our paper to be informative and of value. We have incorporated the helpful feedback that they have provided us to enhance our paper, and have provided responses to each of their comments.

With thanks,

Yusra Shawar

| Reviewer #1 | Our response |
| --- | --- |
| In general, the paper provides rich information to explain why QCN in selected pathfinder countries has emerged differently. I do enjoy reading it even though sometime I get loss due to the paper is so long especially in the result part. if the authors make the result part more concise will be excellent. | Thanks for your positive review of our paper. We agree that the paper is long, and given your comment, have revised our paper down by ~1,500 words in an effort to distill out the main points, without losing the richness of the data. |
| Reviewer #2 | Our response |
| Thank you for the opportunity to review the article entititled, "Factors Shaping Network Emergence: A Cross-Country Comparison of Quality of Care Networks in Bangladesh, Ethiopia, Malawi, and Uganda" I have followed this network for several years and enjoyed reading this detailed narrative and analysis of the QCN implementation. The authors are to be commended for bringing clear lessons and recommendations for future networks. I only have two minor comments: | Thank you for your positive words, especially given your close following of the network over the years. |
| 1. Figure 1 is very blurry and hard to read with the color variation in the boxes and black font. Consider revising. | We have completely revised this figure (and most tables in the paper) for ease of reading. |
| Page 10, Lines 166 to 174 provides great information on the roles of the different organizations. Can authors speak to funding at the country level? Was there a discussion or expectations by the network of how the work was to be funded at country level? I think readers will want to understand that beyond the funding by BMGF for the QCN secretariat and UNICEF. | On page 4, lines 213-218, we have added this important detail about funding expectations at the country level:  “As noted in WHO’s 2021 QCN progress report and further discussed in the QCN meeting in Ghana in March 2023, the expectation was that resources—technical and monetary—for supporting each country network and their broader quality of care improvement initiatives were to be transitioned from global to domestic actors. However, few countries achieved this during the network’ first five years, with exception to Bangladesh and Ethiopia.”  This is also further discussed in papers 2 (Akter et al 2023), 3 (Djellouli et al 2023), and 9 (Lemma et al 2023) in this paper series on QCN, already published in *PloS Global Public Health.* |
